# Characterization of metabolites determined by means of ^1^H HR MAS NMR in intervertebral disc degeneration

**DOI:** 10.1007/s10334-014-0457-0

**Published:** 2014-08-10

**Authors:** Barbara Pacholczyk-Sienicka, Maciej Radek, Andrzej Radek, Stefan Jankowski

**Affiliations:** 1 Faculty of Chemistry, Institute of Organic Chemistry, Łódź University of Technology, Lodz, Poland; 2 Department of Neurosurgery and Peripheral Nerve Surgery, WAM University Hospital, Central Veterans Hospital of the Medical University of Lodz, Lodz, Poland

**Keywords:** Disc degeneration, Metabolic profile, Metabolomics, Quantitative HR MAS NMR, 2-Propanol

## Abstract

**Object:**

The objective of this study is the identification of metabolites by means of ^1^H high resolution magic angle spinning nuclear magnetic resonance (^1^H HR MAS NMR) spectroscopy and the evaluation of their applicability in distinguishing between healthy and degenerated disc tissues.

**Materials and methods:**

Differences between the metabolic profiles of healthy and degenerated disc tissues were studied by means of ^1^H HR MAS NMR. Analysis was performed for 81 disc tissue samples (control samples *n* = 21, degenerated disc tissue samples *n* = 60). Twenty six metabolites (amino acids, carbohydrates, and alcohols) were identified and quantified.

**Results:**

The results indicate that the metabolic profile of degenerated discs is characterized by the presence of 2-propanol and the absence of *scyllo*-inositol and taurine. The concentrations of 2-propanol and lactate increase with age.

**Conclusion:**

PCA analysis of ex vivo ^1^H HR MAS NMR data revealed the occurrence of two groups: healthy and degenerative disc tissues. The effects of insufficient nutrient supply of discs, leading to their degeneration and back pain, are discussed.

**Electronic supplementary material:**

The online version of this article (doi:10.1007/s10334-014-0457-0) contains supplementary material, which is available to authorized users.

## Introduction

Intervertebral disc degeneration (IVDD) is a common clinical problem whose pathogenesis is still not very well understood. However, it is known that IVDD causes biochemical and morphological changes in the structure of the disc, leading to the deterioration of the biomechanical function of the joint and low back pain [1–4]. Disc degeneration may result from mechanical trauma [3, 5], patient age, familial predisposition [5, 6], and an imbalance between anabolic and catabolic processes [7]. A healthy disc consists of three elements: the nucleus pulposus (NP), the annulus fibrosus (AF), and endplates separating the nucleus from the adjacent vertebral bone [1]. The central region of the nucleus pulposus is highly hydrated and acts as a hydraulic cushion to withstand the forces of compression and torsion. The annulus fibrosus prevents the nucleus pulposus from herniating or leaking out of the disc by hydraulically sealing the nucleus and evenly distributing any pressure and force imposed on the intervertebral disc. Radiography, discography, computed tomography (CT), and magnetic resonance imaging (MRI) are used to diagnose disc degeneration [8–13]. Interpreting the results of imaging is difficult and complicated because discs are composed of several subtissues (nucleus, annulus, endplate) and there is poor correlation between morphological findings, spinal biomechanics, and patient symptoms [3, 14–17].

High resolution magic angle spinning (HR MAS) ^1^H NMR spectroscopy is a nondestructive technique that has been applied to characterize the composition of various intact human cancer tissues, such as breast [18, 19], brain [20, 21], prostate [22–24], lung [25], and colon [26] cancers. This technique has also been applied to determine the biomarkers of disc degeneration. Keshari et al. [1–3] observed changes in the chemical composition of discs, especially increased levels of unbound hydroxyproline and glycine, associated with collagen breakdown. They noticed a decrease in the concentration of proteoglycans correlated with degeneration and suggested that lactate, collagen, and proteoglycan may serve as metabolic markers in discogenic back pain.

A synergic combination of NMR spectroscopy and chemometric techniques offers a useful tool for the identification of markers of degenerative disc disease.

The purpose of this study is the determination of disc metabolites in order to evaluate their applicability in the diagnosis of intervertebral disc degeneration by means of ^1^H HR MAS NMR. We hope that in the near future HR MAS NMR ex vivo studies of metabolic profiles combined with in vivo studies using MRI scanners (MRS) may become part of a new diagnostic protocol.

## Materials and methods

### Disc tissue samples

Analysis was performed for 81 disc tissue samples (control samples *n* = 21, degenerated disc tissue samples *n* = 60). Tissue samples were harvested from 60 patients who underwent intervertebral disc surgery at the WAM University Hospital, Central Veteran Hospital of the Medical University of Łódź, Poland. The reference samples originated from non-degenerated discs after mechanical trauma (*n* = 20) and from a post-mortem section (*n* = 1). This project was approved by the local ethics committee (Approval No. RNN/355/12/KB). The patients were diagnosed on the basis of CT and MRI. The specimens were collected during classic microdiscectomy from the cervical and lumbar spinal regions. After standard preparation of the operation field, intervertebral disc exposure was performed using microsurgery. The annulus fibrosus was fenestrated under the operating microscope and a 3 mm × 3 mm specimen was taken. The harvested fragment was denoted as annulus. Afterwards, tissue was harvested from the deeper layers of the intervertebral disc and denoted as nucleus. For each patient, annulus fibrosus and nucleus pulposus samples were analyzed by means of ^1^H HR MAS NMR. Analysis was performed on the following lumbar (L) and cervical (C) intervertebral discs: L1/L2 (*n* = 2), L2/L3 (*n* = 2), L3/L4 (*n* = 3), L4/L5 (*n* = 20), L5/S1 (*n* = 30), C2/C3 (*n* = 2), C5/C6 (*n* = 12), and C6/C7 (*n* = 10).

All tissue samples were placed on ice after surgery for 15 min and then stored at −80 °C until HR MAS analysis within 1 week. All samples were treated in the same way. No changes in ^1^H NMR spectra were observed after 7 days in storage at −80 °C temperature. Prior to HR MAS analysis, all samples were cut to fit the 4 mm zirconium HR MAS rotor (a total sample volume of 50 μL). Samples weighed 37.29 mg on average (range 5.31–60.49 mg). The mean age of the patients was 46.5 ± 13.7 (SD) years (range 16–78 years).

### HR MAS experiments

All spectra were acquired using a Bruker Avance II Plus 16.4 T spectrometer (BrukerBioSpin, Germany) operating atan ^1^H frequency (700.08 MHz). The instrument was equipped with a 4 mm ^1^H/^13^C HR MAS probe with the gradient aligned along the magic angle axis. Samples were spun at 6 kHz to keep rotation sidebands out of the acquisition window. All experiments were conducted at nominally 25 °C for 20 min, to avoid the TCA cycle metabolites such as succinate, citrate, and oxaloacetate formation. Changes of the concentration of the other metabolites were not observed during this short time. At lower temperatures (5 and 15 °C) the resolution of the ^1^H spectra was changed for the worse. Phosphate-buffered saline (PBS 0.1 M, pH 7.4, 30 μL) made with deuterium oxide and containing 3.8 mM TSP (sodium-3′-trimethylsilylpropionate-2,2,3,3-d_4_) was added to the each sample. A Carr–Purcell–Meiboom–Gill (CPMGpr, Bruker) spin-echo sequence [27] was applied with a delay of 1 ms, repeated 140 times. Spectra were recorded with 1.5 s water presaturation during the relaxation delay, and a calibrated 90° pulse was applied for 128 scans, collecting 64 K data points over a spectral width of 14,097 Hz. The repetition time of 10.32 s, including a relaxation delay of 8 s, was calculated as 7T_1_, which had the longest relaxation time to ensure complete magnetization recovery. An exponential line broadening of 0.30 Hz was applied to raw data prior to Fourier transformation. The TSP peak at 0 ppm was used as a chemical shift standard and a linear baseline correction was applied. Homonuclear correlated spectra (COSY) and total correlation spectroscopy (TOCSY) experiments were performed on selected samples to aid peak identification. Homonuclear correlated spectra (COSY) were acquired using a standard pulse sequence [28]. Spectra were recorded with acquisition of 16 transients for each of the 512 increments with 2 K data points. TOCSY was acquired using a standard pulse sequence [29]. Mixing time was 80 ms. In spectra recorded for 20 min, only correlations for highly concentrated metabolites were observed.

### Quantification of metabolites in disc samples

Quantitative analysis was performed for the spectral regions from 0.8 to 4.65, from 5.0 to 8.2, and the region corresponding to TSP (from 0.1 to −0.1 ppm). The signal of water (4.66–5.0) was not analyzed. All spectral regions were individually corrected using a fifth-order baseline function. Molar metabolite concentrations were calculated from the equation:

$$[{\text{MET}}] = \frac{{A_{\text{MET}} }}{{A_{\text{TSP}} }} \times \frac{{H_{\text{TSP}} }}{{H_{\text{MET}} }} \times \frac{{n_{\text{TSP}} }}{{m_{\text{sample}} }},$$

where *A*_MET_ and *A*_TSP_ are areas of metabolite and TSP signals, respectively; *H*_MET_ and *H*_TSP_ are the numbers of protons per metabolite and TSP signals; *n*_TSP_ is the number of moles the TSP signal represents, and *m*_sample_ is the weight of the sample in the MAS rotor.

### Statistical analysis

Statistical analysis was performed using AMIX 3.9.14 (Bruker, Germany). The variation of the data was explored by principal component analysis (PCA), with NP spectra being more suitable for this type of analysis. The spectral region from 3.29 to 4.05 of spin-echo ^1^H NMR spectra was chosen as input data for PCA analysis. Linear combinations of metabolites that explain most of the overall variance in the data set were calculated by means of PCA. Baseline offset was corrected, and the selected spectral region was mean-normalized to arrive at the total area for each sample.

## Results

HR MAS NMR spectroscopy makes it possible to determine metabolite concentrations in intact tissues. Sample preparation for HR MAS is simpler than analysis of extracts, which requires sample homogenization and standardization for each metabolite. The extraction process is laborious, time-consuming, and destructive [30].

**Fig. 1:**
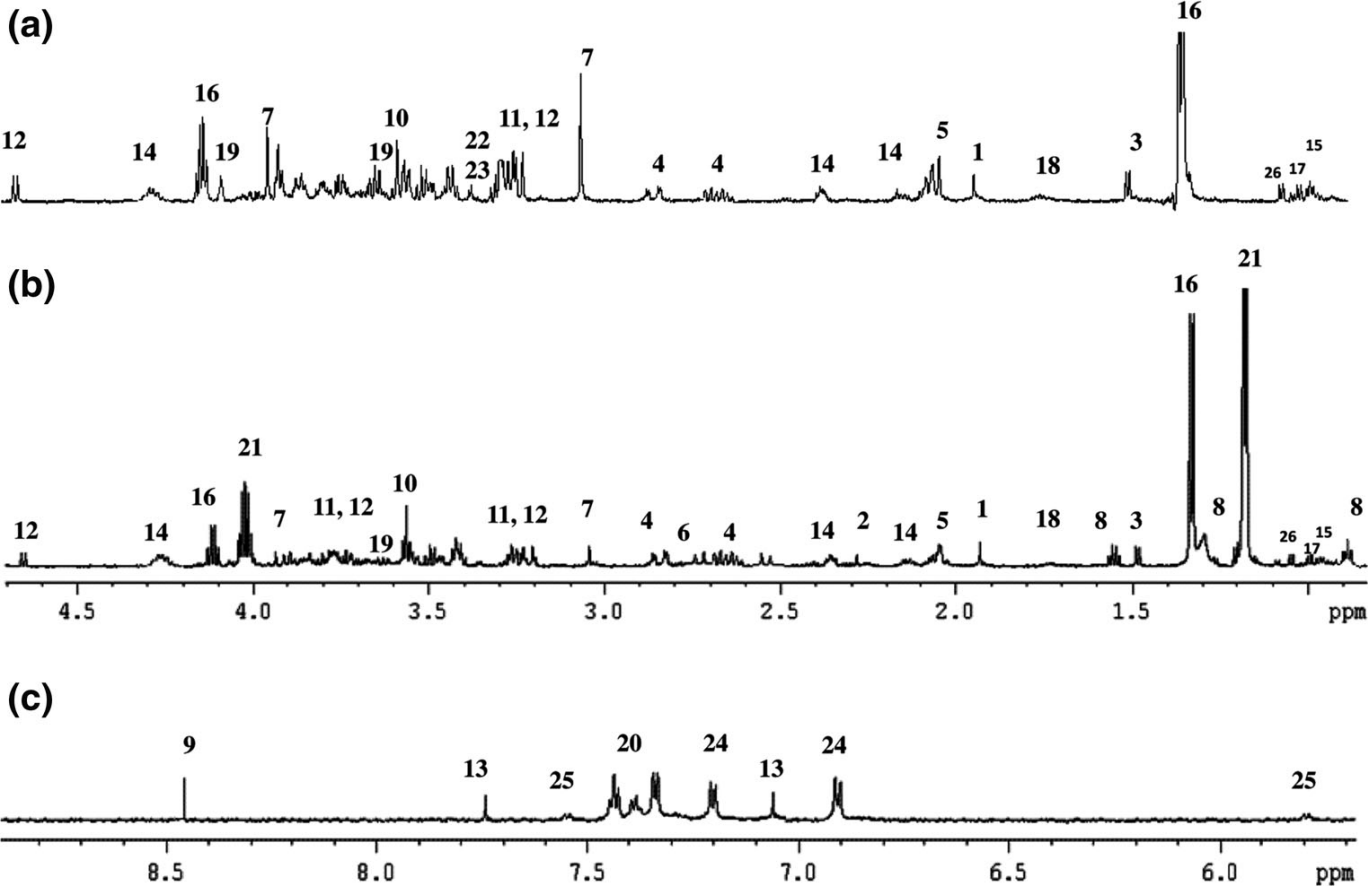
Representative ex vivo ^1^H HR MAS NMR spectra of a healthy disc (**a**), and of a degenerated disc for the aliphatic (**b**) and aromatic (**c**) regions of the spectrum

Representative ex vivo ^1^H HR MASNMR spectra of healthy and degenerated disc tissues are shown in Fig. 1. Assignment of metabolite resonances was based on analysis of one dimensional ^1^H, two dimensional correlation spectroscopy (COSY), and total correlation spectroscopy (TOCSY) NMR spectra. The collected data were compared with those from the literature [25] and with the spectra of metabolites recorded on a Bruker Avance II Plus700 MHz spectrometer. The^1^H HR MAS NMR spectra for nucleus pulposus and annulus fibrosus specimens were very similar (see Figure SM1 in supplementary material). In the HR MAS spectra of disc tissues, signals of five groups of compounds were observed. The main metabolites characterizing disc tissues are labeled in Fig. 1 and the pool of metabolites is reported in Table 1. The largest group consisted of 12 amino acids, which were derived from protein cores and were also formed by collagen breakdown. The second group of compounds was comprised of carboxylic acids and their derivatives, mainly lactic acid, citric acid, formate, acetate, and acetone. Moreover, some carbohydrates (mainly α- and β-glucose and chondroitin sulfate) and pyrimidine derivatives (e.g. uracil) were found in the spectra of disc tissues. Alcohols, such as *myo*-inositol, *scyllo*-inositol, and 2-propanol, were also identified. In the case of 2-propanol, the possibility of its exogenous origin was checked at every step from surgery to HR MAS measurements, but it was deemed possible for 2-propanol to have been introduced into disc tissue samples as a result of its antiseptic use. The patients were being administered pain relief and non-steroidal anti-inflammatory drugs, but 2-propanol was not found to be a component or metabolite of those drugs. Resonances of 2-propanol was observed due to good resolution of ^1^H NMR spectra and confirmed by COSY/TOCSY experiments (see Figure SM3–SM4 in supplementary materials). The unassigned signal at 1.2 ppm reported earlier [2] probably corresponds to 2-propanol, but was not recognized in poorly resolved spectra.

**Table 1:** Metabolites assigned in HR MAS spectra of disc tissues, their diagnostic ^1^H signals, chemical shifts (*δ*_H)_ and multiplicities

Assignment number	Metabolite	Assignment	^1^H multiplicity	*δ*_H_, ppm
1	Acetate	CH_3_	s	1.93
2	Acetone	CH_3_	s	2.23
3	Alanine	CH_3_	d	1.48
		CH	q	3.78
4	Aspartate	β-CH_2_	dd, dd	2.67, 2.81
		α-CH	dd	3.91
5	Chondroitin sulfate	*N*-acetyl	s	2.05
6	Citric acid	CH_2_	d	2.54
		CH_2_	d	2.66
7	Creatine	CH_3_	s	3.03
		CH_2_	s	3.93
8	Fatty acids	CH_3_	t	0.90
		(CH_2_)_n_	m	1.29
		CH_2_–CH_2_–CO	q	1.55
9	Formate		s	8.45
10	Glycine	α-CH_2_	s	3.55
11	α-Glucose	C4H		3.40
		C2H	dd	3.54
		C3H	dd	3.71
		C6H		3.83
		C5H		3.85
		C1H	d	5.23
12	β-Glucose	C2H	dd	3.24
		C4H		3.41
		C5H	dd	3.46
		C3H	t	3.49
		C6H	t	3.76
		C6′H	dd	3.90
		C1H	d	4.64
13	Histidine	C2H, ring	s	7.05
		C4H,ring	s	7.75
14	Hydroxyproline	β-CH	m	2.13
		β′-CH	m	2.36
		δ-CH	dt	3.35
		δ′-CH	dd	3.42
		α-CH	dd	4.26
		γ-CH	t	4.67
15	Isoleucine	δ-CH_3_	t	0.94
		γ-CH_3_	d	1.01
		γ-CH_2_	m	1.27–1.47
		β-CH	m	1.98
		α-CH	d	3.67
16	Lactate	CH_3_	d	1.33
		CH	q	4.11
17	Leucine	δ-CH_3_	d	0.96
		δ-CH_3_	d	0.97
		γ-CH	m	1.70
		β-CH_2_	m	1.72
		α-CH	m	3.74
18	Lysine	δ-CH_2_	m	1.41
		γ-CH_2_	m	1.67
		β-CH_2_	m	1.70
		ε-CH_2_	t	3.02
		α-CH	t	3.77
19	*myo*-Inositol	C5H	t	3.27
		C1H, C3H	dd	3.53
		C4H, C6H	t	3.62
		C2H	t	4.06
20	Phenylalanine	β-CH	dd	3.21
		α-CH	dd	3.97
		C2H, C6H, ring	m	7.33
		C4H, ring	m	7.38
		C3H, C5H, ring	m	7.43
21	2-Propanol	CH_3_	d	1.17
		CH	sp	4.02
22	*scyllo*-Inositol	CH	s	3.35
23	Taurine	S-CH_2_	t	3.29
		N-CH_2_	t	3.43
24	Tyrosine	β-CH	dd	3.20
		β′-CH	dd	3.05
		α-CH	dd	3.94
		C3H, C5H, ring	d	6.91
		C2H, C6H, ring	d	7.19
25	Uracil	5-CH, ring	d	5.80
		6-CH, ring	d	7.53
26	Valine	γ-CH_3_	d	1.00
		γ-CH_3_	d	1.04
		β-CH	m	2.27
		α-CH	d	3.61

*Notation*: *s* singlet, *d* dublet, *dd* dublet of dublets, *t* triplet, *dt* dublet of triplets, *sp* septet, *q* quartet, *m* multiplet

**Fig. 2:**
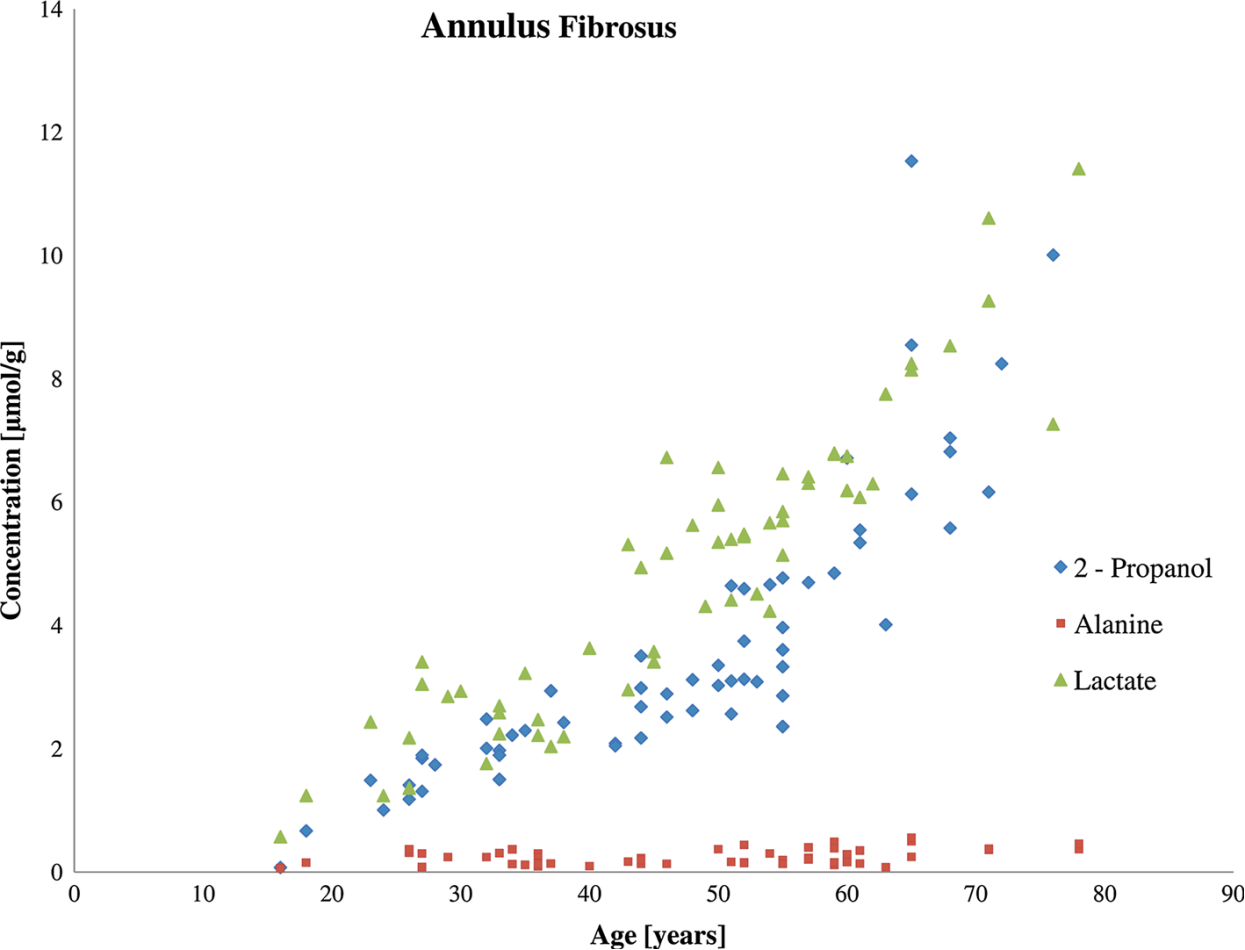
Relationship between patient age and the concentrations of 2-propanol, lactate, and alanine in the annulus fibrosus of degenerated discs

**Fig. 3:**
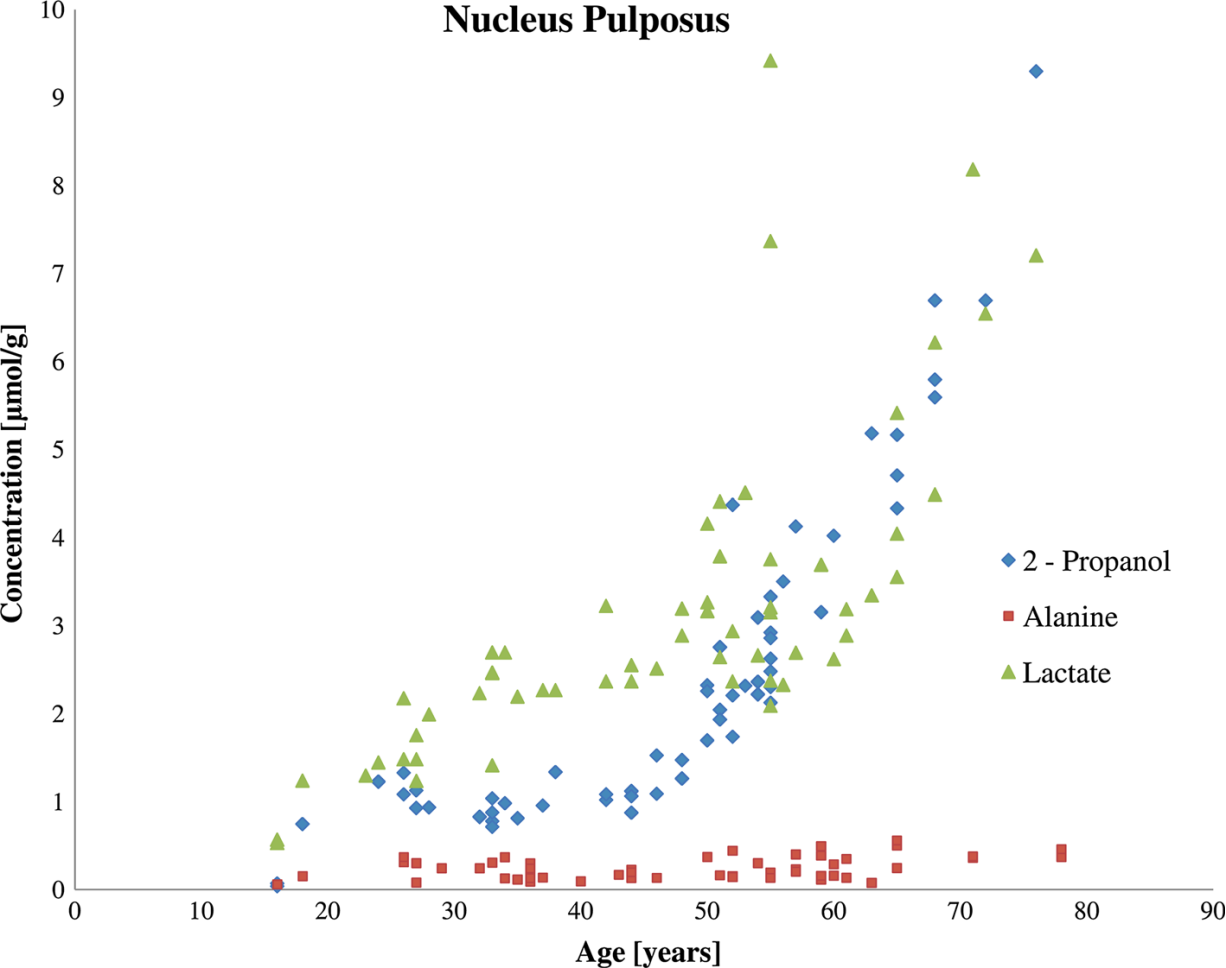
Relationship between age and the concentrations of 2-propanol, lactate, and alanine in the nucleus pulposus of degenerated discs

The presented study revealed that in degenerated disc samples, the concentrations of 2-propanol and lactate in the nucleus pulposus and the annulus fibrosus increase significantly with age, whereas the concentrations of other metabolites do not change significantly with age. As an example, alanine was shown in Figs. 2 and 3. The concentrations of these compounds are elevated in degenerated discs, particularly in patients above the age of 45. Linear curve fitting (0–45 and 45–90) was performed to reveal the age effect on concentrations of 2-propanol and lactate. For annulus fibrosous the slope of the curve increased from 0.095 to 0.31 for 2-propanol, and from 0.093 to 0.18 for lactate. For the nucleus pulposus analogue correlation showed an increase of the curve slope from 0.019 to 0.27 for 2-propanol, and from 0.089 to 0.18 for lactate. There is no correlation between age and concentration of lactate in control samples. These correlations are presented in detail in Figures SM5–SM9 in the supplementary materials.

**Fig. 4:**
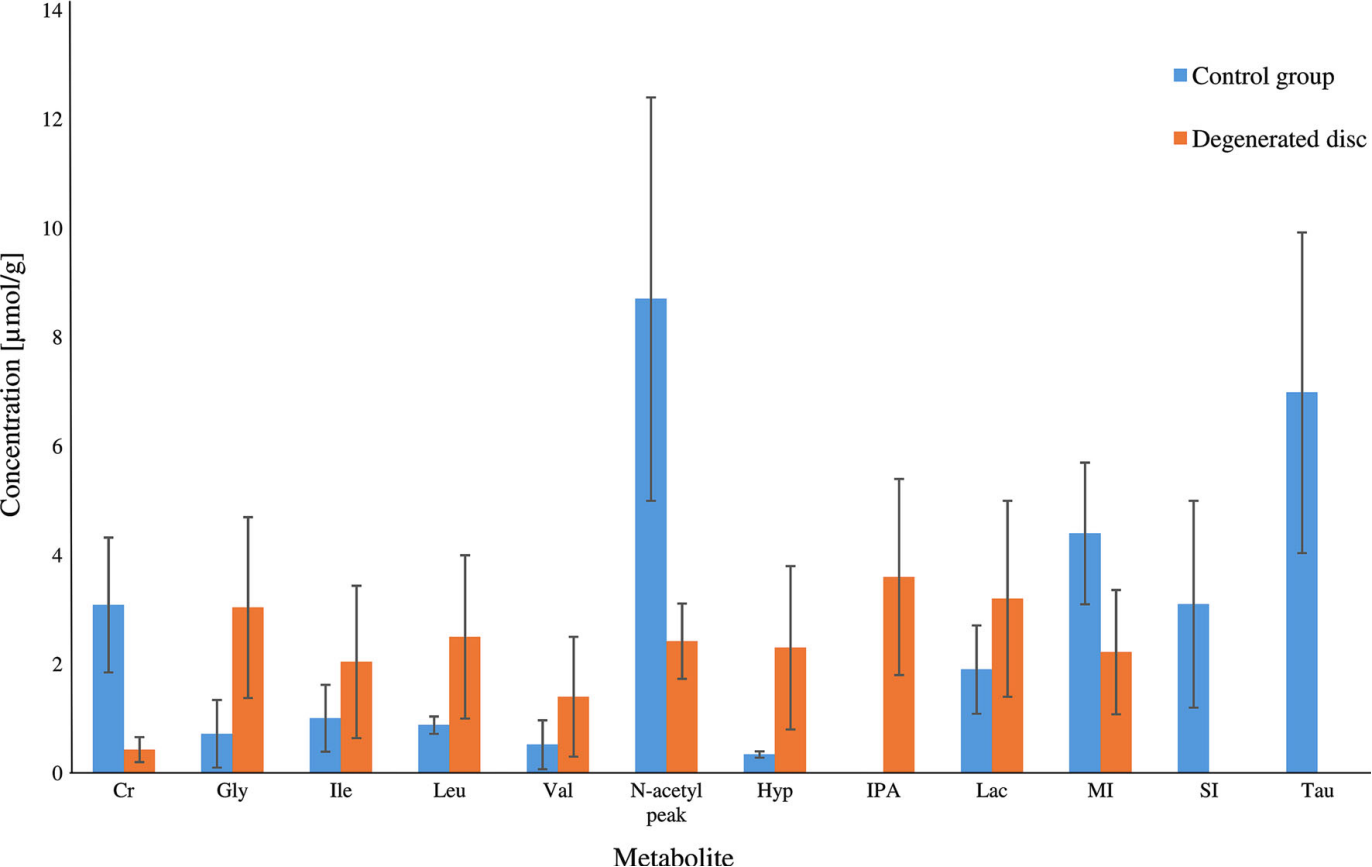
Concentrations of metabolites in degenerated discs and the control group. Other metabolites are reported as Supplementary material in Tables SM1–SM3 and Figure SM2

Metabolic changes in degenerative disc disease were also observed based on analysis of the main components of intervertebral discs: proteoglycans and glycosaminoglycans. The degradation of proteoglycans was observed in ^1^H HR MAS spectra as an increase in amino acid levels, mainly glycine, hydroxyproline, isoleucine, leucine, and valine. These relationships are shown in Fig. 4. Due to the decomposition of glycosaminoglycans (mainly chondroitin sulfate), the intensity of the *N*-acetyl peak in the proton spectra of the nucleus pulposus and the annulus fibrosus decreased with patient age.

### Principal component analysis

**Fig. 5:**
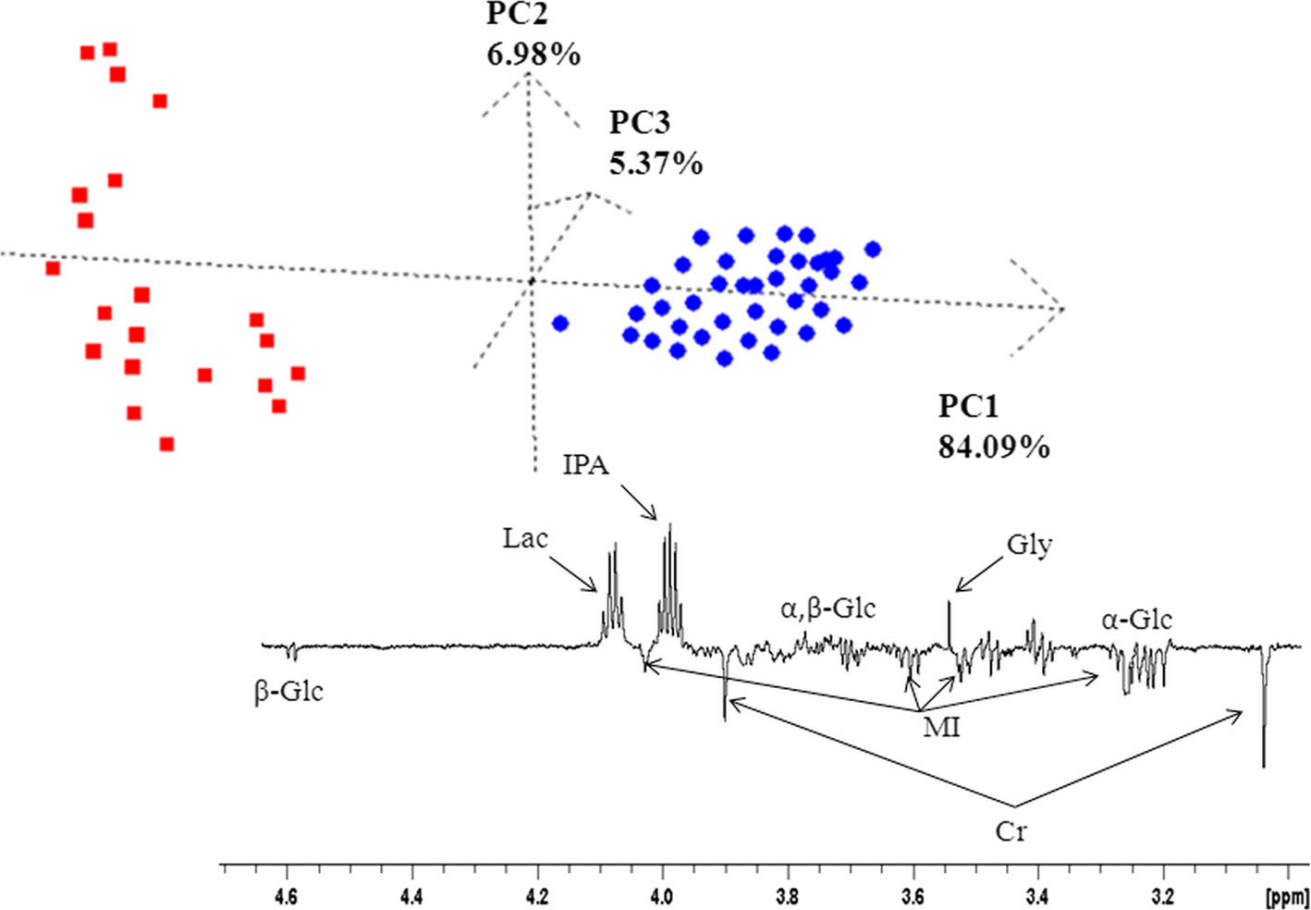
A score plot of PC1 versus PC2 versus PC3 and a loading plot of PC1 from PCA of spin-echo spectra from patients diagnosed with intervertebral disc degeneration (*circles*) and from healthy samples (*squares*)

PCA explains the variance in structure of a set of variables through linear combinations of the variables (principal components, PCs). The obtained PCA score plot distinguished between degenerated disc tissue samples and healthy tissues, based on selected signals of protons detected by ^1^H HR MAS NMR. Analysis of the scores of PC1 versus PC2 versus PC3 (describing 96.06 % of total variation) led to two distinct groups. One consists of degenerated disc samples, while the other one is healthy disc samples. The metabolic profile of the healthy samples is characterized by the absence of 2-propanol and the presence of *scyllo*-inositol and taurine at the 3.29–4.05 ppm level. Moreover, a higher level of glycine and lower concentrations of *myo*-inositol, creatine, and glucose were observed in degenerated discs than in healthy tissues. A PCA score plot based on the spin-echo spectra of 60 samples is shown in Fig. 5.

## Discussion

Intervertebral disc cells play an integral and vital role in maintaining disc health and function. The composition and degradation of IVD tissue is controlled by the cells, because they synthesize the extracellular matrix of the discs as well as matrix metalloproteinases (MMPs), which are responsible for matrix breakdown [31]. Healthy tissues are characterized by a balance between matrix production and degradation. When this balance is disrupted, the disc matrix can be changed. Alteration in disc cell metabolism may lead to changes in disc structure and composition. Nutrient and oxygen diffusion across the IVD matrix, soluble regulators of cell function, genetic influences, ageing, and mechanical load are known to be major factors affecting disc function [32].

NMR spectra of non-degenerated and degenerated disc tissues revealed that they had different metabolic profiles. In particular, taurine and *scyllo*-inositol were observed only in healthy disc tissues, whereas *myo*-inositol was found only in small amounts in degenerated discs. *myo*-Inositol and *scyllo*-inositol are important osmolytes responsible for the regulation of long term hypoosmotic and hyperosmotic stress. They ensure osmotic equilibrium between cells and the surrounding tissues. Changes in the concentrations of these three metabolites may indicate an imbalance in the osmolyte function of discs in degenerative diseases. *myo*-Inositol is crucial for the optimal functioning of neurons, and alteration of its concentration leads to disturbances in the physiological properties of nerves [33]. Taurine plays an important role in short-term hypoosmotic stress [34–36]. It also serves as a neurotransmitter in the brain, an antioxidant, and a facilitator in the transport of ions such as sodium, calcium, potassium, and magnesium. Taurine deficiency has been observed in a variety of diseases [37–39].

In addition, a lower concentration of creatine and elevated concentrations of glycine and hydroxyproline were observed in degenerated discs. Increased concentrations of glycine and hydroxyproline in degenerated discs have been observed previously, and have been associated with collagen breakdown [1–3]. Lower concentrations of creatine in degenerated discs might result from disturbed cell energy metabolism. Creatine plays a pivotal role in the energy metabolism of cells. This amino acid acts as an “ATP shuttle,” carrying ATP to the sites where it is utilized [40].

The observed elevated 2-propanol level may originate from a deficiency of disc nutrition. During anaerobic glycolysis, disc cells obtain their energy in the form of adenosine triphosphate (ATP), which is produced during quantitative conversion of glucose to lactic acid. An accumulation of lactic acid in the tissues causes acidification (pH < 6.4), which may lead to cell death. Furthermore, low glucose concentrations (below 0.5 mM) persisting for more than approximately 3 days may also result in cell death [41, 42]. Under slightly less acidic conditions (pH < 6.8), the rate of matrix production decreases while the rate of matrix degradation remains unchanged, causing an imbalance favoring matrix breakdown [43]. The lower pH resulting from increased lactic acid levels enhances the enzymatic activity of metalloproteinases, which in turn causes the degradation of collagen into its amino acid constituents [44]. The intervertebral disc is the largest avascular tissue in the body, and thus nutritional supply to disc tissue may occur either by diffusion of small molecules such as glucose or by convection of larger molecules [45–47]. Dehydration and the related disc degeneration cause changes in disc structure and may constrain the transport of nutrients. A lack of sufficient cellular nutrition may lead to new ways of glucose delivery, for instance, by fatty acid metabolism. The main product of this metabolism is acetyl-CoA, which is precipitated under certain conditions such as starvation, chronic alcoholism, or high fat intake. Acetoacetate is formed by coupling two molecules of acetyl-CoA, which then undergo spontaneous decarboxylation to acetone. Acetone in the presence of elevated NADH/NAD^+^ ratios in reactions catalyzed by alcohol dehydrogenase is reduced to 2-propanol [48].

The presence of 2-propanol has been reported previously in diabetic ketoacidosis, hypothermia [49], starvation, dehydration, chronic ethanol use [50], breast cancer [51], and in exhaled breath from smokers [52].

## Conclusion

HR MAS analysis of intervertebral discs provided new insights into the degenerative disease process. PCA of ex vivo ^1^H HR MAS NMR data distinguished between two groups: healthy and degenerated disc tissues. Our results indicate that the metabolic profile of degenerated discs is characterized by the presence of 2-propanol and the absence of *scyllo*-inositol and taurine. Moreover, a decrease in creatine and *myo*-inositol concentrations was observed in degenerated discs. The concentrations of 2-propanol and lactate increase with age.

## Electronic supplementary material

Below is the link to the electronic supplementary material.

Supplementary material 1 (DOCX 29 kb)

Supplementary material 2 (TIFF 1384 kb)

Supplementary material 3 (TIFF 91 kb)

Supplementary material 4 (DOCX 154 kb)

Supplementary material 5 (DOCX 15 kb)

Supplementary material 6 (DOCX 17 kb)

Supplementary material 7 (DOCX 15 kb)

## Abbreviations

Ac: Acetate
Ala: Alanine
Asp: Aspartate
AF: Annulus fibrosus
ADH: Alcohol dehydrogenase
ATP: Adenosine-5′-triphosphate
Cit: Citrate
CoA: Coenzyme A
COSY: Correlation spectroscopy
Cr: Creatine
CT: Computed tomography
GAG: Glycosaminoglycans
Glc: Glucose
Gly: Glycine
His: Histidine
Hyp: Hydroxyproline
HR MAS: High-resolution magic angle spinning
Ile: Isoleucine
IPA: 2-Propanol
IVDD: Intervertebral disc degeneration
Lac: Lactate
Leu: Leucine
Lys: Lysine
*N*-acetyl peak: Chondroitin sulfate
NAD^+^: Nicotinamide adenine dinucleotide
NP: Nucleus pulposus
MI: *myo*-Inositol
MMPs: Matrix metalloproteinases
MRI: Magnetic resonance imaging
MRS: Magnetic resonance spectroscopy
PBS: Phosphate buffered saline
PCA: Principal component analysis
PG: Proteoglycans
Phe: Phenyloalanine
SD: Standard deviation
SI: *scyllo*-Inositol
Suc: Succinate
Tau: Taurine
TCA: Tricarboxylic acid cycle
TOCSY: Total correlation spectroscopy
TSP: Trimethylsilyltetradeuteropropionic acid
Tyr: Tyrosine
U: Uracil
Val: Valine
