# Supplementary material for: Characterization of metabolites determined by means of 1H HR MAS NMR in intervertebral disc degeneration
Source: MAGMA. 2014 Aug 10;28(2):173–83. doi: 10.1007/s10334-014-0457-0 (PMC4385564; doi:10.1007/s10334-014-0457-0)
Supplement: Supplementary file 1 — Supplementary material 1 (DOCX 29 kb) [file 10334_2014_457_MOESM1_ESM.docx]

CHARACTERIZATION OF THE METABOLITES IN INTERVERTEBRAL DISC DEGENERATION DETERMINED BY ^1^H HR MAS NMR SPECTROSCOPY

Magnetic Resonance Materials in Physics Biology and Medicine

**Barbara Pacholczyk - Sienicka^a^, Maciej Radek^b^, Andrzej Radek^b^ and Stefan Jankowski^a*^**

^a^*Institute of Organic Chemistry, Faculty of Chemistry, Łódź University of Technology, Poland*

**^b^***Department of Neurosurgery and Peripheral Nerve Surgery, WAM University Hospital, Central Veterans Hospital of Medical University of Łódź, Poland*

*Corresponding author. Tel: +48-42-631 3222; e-mail: [stefan.jankowski@p.lodz.pl](mailto:stefan.jankowski@p.lodz.pl)

Fig. SM5. Linear curve fitting performed for 2- propanol for nucleus pulposus tissues of degenerated discs.

Fig. SM6. Linear curve fitting performed for lactate for nucleus pulposus tissues of degenerated discs.

Fig. SM7. Linear curve fitting performed for 2- propanol for annulus fibrosus tissues of degenerated discs.

Fig. SM8. Linear curve fitting performed for lactate for annulus fibrosus tissues of degenerated discs.

Fig. SM9. Linear curve fitting performed for lactate for nucleus pulposus tissues of control discs.
