# Supplementary figures and images for: Characterization of metabolites determined by means of 1H HR MAS NMR in intervertebral disc degeneration
Source: MAGMA. 2014 Aug 10;28(2):173–83. doi: 10.1007/s10334-014-0457-0 (PMC4385564; doi:10.1007/s10334-014-0457-0)

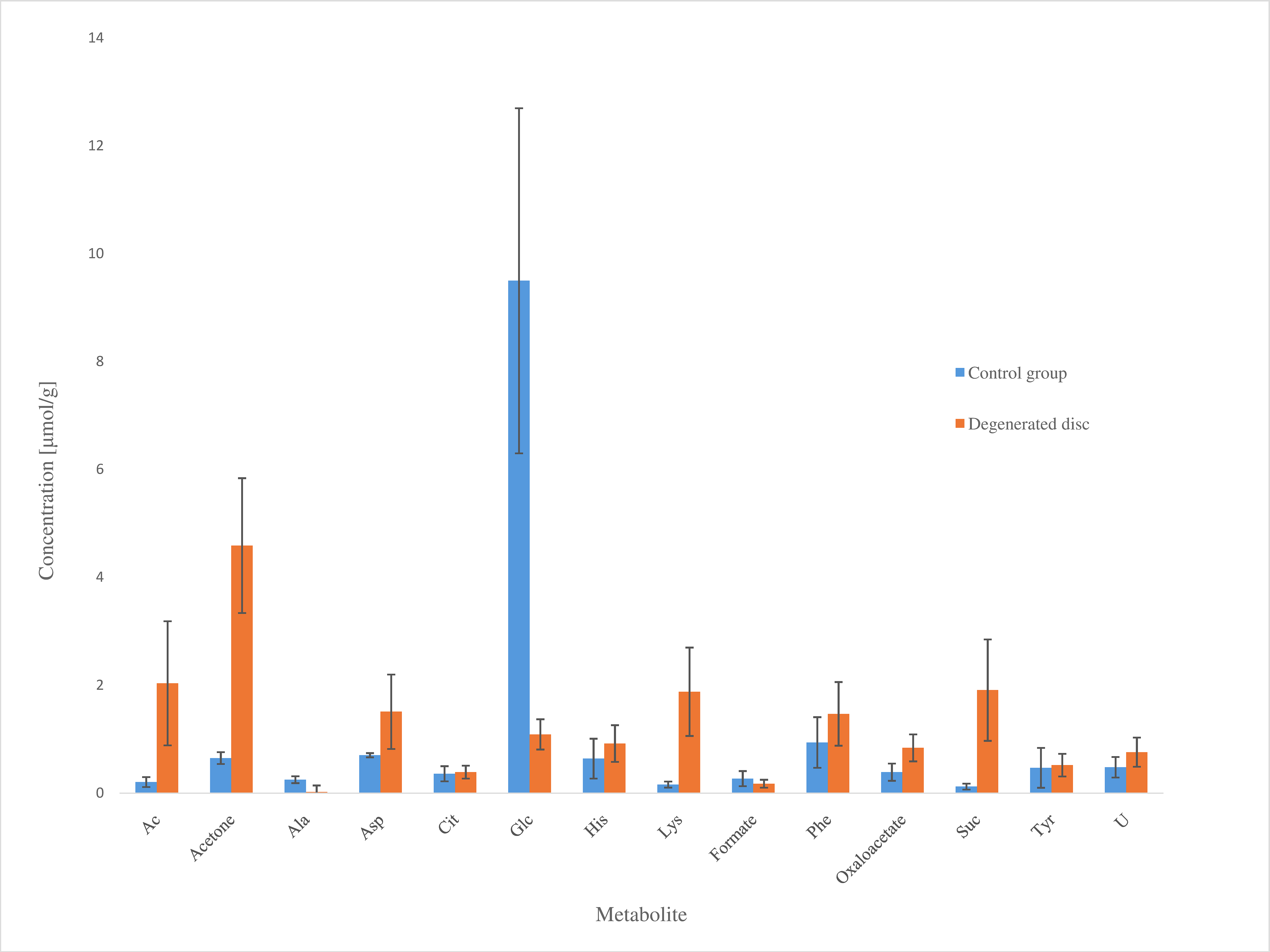

Supplement: Supplementary file 2 — Supplementary material 2 (TIFF 1384 kb) [file 10334_2014_457_MOESM2_ESM.tif]

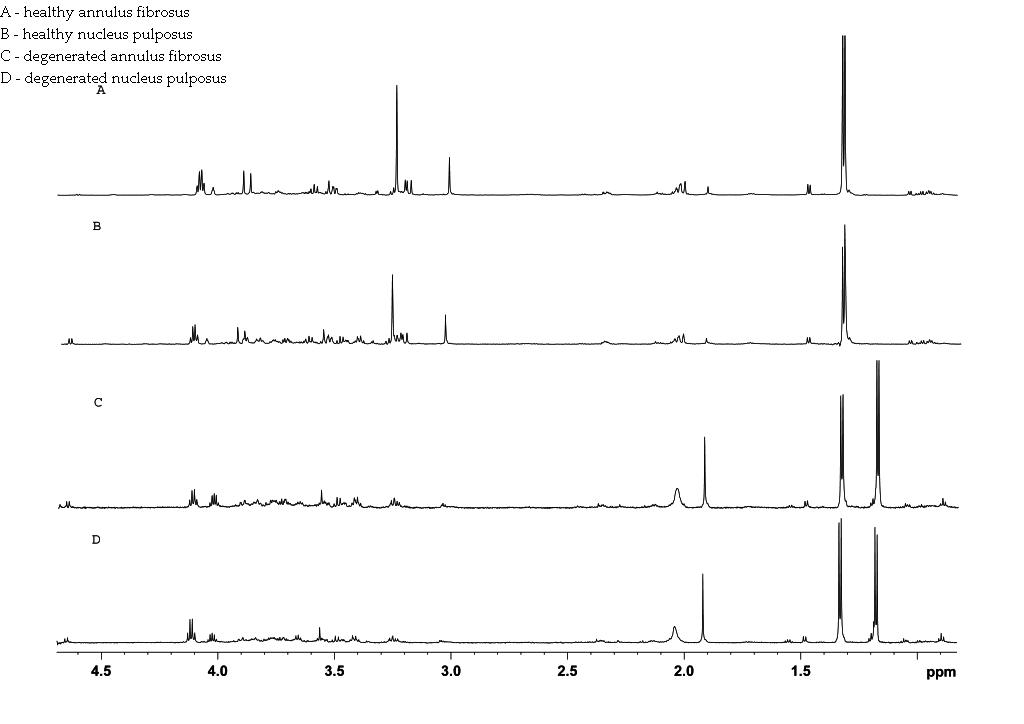

Supplement: Supplementary file 3 — Supplementary material 3 (TIFF 91 kb) [file 10334_2014_457_MOESM3_ESM.tif]
