# Supplementary material for: Characterization of metabolites determined by means of 1H HR MAS NMR in intervertebral disc degeneration
Source: MAGMA. 2014 Aug 10;28(2):173–83. doi: 10.1007/s10334-014-0457-0 (PMC4385564; doi:10.1007/s10334-014-0457-0)
Supplement: Supplementary file 4 — Supplementary material 4 (DOCX 154 kb) [file 10334_2014_457_MOESM4_ESM.docx]

CHARACTERIZATION OF THE METABOLITES IN INTERVERTEBRAL DISC DEGENERATION DETERMINED BY ^1^H HR MAS NMR SPECTROSCOPY

Magnetic Resonance Materials in Physics Biology and Medicine

**Barbara Pacholczyk - Sienicka^a^, Maciej Radek^b^, Andrzej Radek^b^ and Stefan Jankowski^a*^**

^a^*Institute of Organic Chemistry, Faculty of Chemistry, Łódź University of Technology, Poland*

**^b^***Department of Neurosurgery and Peripheral Nerve Surgery, WAM University Hospital, Central Veterans Hospital of Medical University of Łódź, Poland*

*Corresponding author. Tel: +48-42-631 3222; e-mail: [stefan.jankowski@p.lodz.pl](mailto:stefan.jankowski@p.lodz.pl)

Fig. SM3. COSY spectrum recorded on Bruker Avance II Plus 700 MHz. Time of acquisition was 20 minutes. Correlations for highly concentrated metabolites: 2-propanol and lactate were observed.

Fig. SM4. COSY spectrum recorded on Bruker Avance II Plus 700 MHz. Time of acquisition was 120 minutes.
