# Supplementary material for: Characterization of metabolites determined by means of 1H HR MAS NMR in intervertebral disc degeneration
Source: MAGMA. 2014 Aug 10;28(2):173–83. doi: 10.1007/s10334-014-0457-0 (PMC4385564; doi:10.1007/s10334-014-0457-0)
Supplement: Supplementary file 5 — Supplementary material 5 (DOCX 15 kb) [file 10334_2014_457_MOESM5_ESM.docx]

Supplementary material for article “Characterization of the metabolites in intervertebral disc degeneration determined by ^1^H HR MAS NMR spectroscopy” by Barbara Pacholczyk - Sienicka, Maciej Radek, Andrzej Radek and Stefan Jankowski.

The additional details of metabolites concentration, linear curve fitting for 2– propanol and lactate are attached. Moreover COSY spectra and comparison of healthy and degenerated ^1^H HR MAS spectra for nucleus pulposus and annulus fibrosus are provided in Figures SM1-SM4.

The legends for the supplementary figures:

1. Figure SM1. Comparison of healthy and degenerated ^1^H HR MAS spectra for nucleus pulposus and annulus fibrosus.
2. Figure SM2. Concentrations of metabolites in degenerated discs and the control group.
3. Figure SM3. COSY spectrum recorded on Bruker Avance II Plus 700 MHz. Time of acquisition was 20 minutes. Correlations for highly concentrated metabolites: 2-propanol and lactate were observed.
4. Figure SM4. COSY spectrum recorded on Bruker Avance II Plus 700 MHz. Time of acquisition was 120 minutes.
5. Figure SM5. Linear curve fitting performed for 2- propanol for nucleus pulposus tissues of degenerated discs.
6. Figure SM6. Linear curve fitting performed for lactate for nucleus pulposus tissues of degenerated discs.
7. Figure SM7. Linear curve fitting performed for 2- propanol for annulus fibrosus tissues of degenerated discs.
8. Figure SM8. Linear curve fitting performed for lactate for annulus fibrosus tissues of degenerated discs.
9. Figure SM9. Linear curve fitting performed for lactate for nucleus pulposus tissues of control discs.

The legends for the supplementary tables:

1. Table SM1. Concentrations of metabolites (μmol/g) in AF and NP (control disc, 14 years old male, L5/S1).
2. Table SM2. Concentrations of metabolites (μmol/g) in AF and NP **(**degenerated disc, 46 years old female, L5/S1).
3. Table SM3. Mean concentrations of metabolites [μmol/g] with standard deviation in intervertebral disc tissues.
