# Supplementary material for: Characterization of metabolites determined by means of 1H HR MAS NMR in intervertebral disc degeneration
Source: MAGMA. 2014 Aug 10;28(2):173–83. doi: 10.1007/s10334-014-0457-0 (PMC4385564; doi:10.1007/s10334-014-0457-0)
Supplement: Supplementary file 6 — Supplementary material 6 (DOCX 17 kb) [file 10334_2014_457_MOESM6_ESM.docx]

CHARACTERIZATION OF THE METABOLITES IN INTERVERTEBRAL DISC DEGENERATION DETERMINED BY ^1^H HR MAS NMR SPECTROSCOPY

Magnetic Resonance Materials in Physics Biology and Medicine

**Barbara Pacholczyk - Sienicka^a^, Maciej Radek^b^, Andrzej Radek^b^ and Stefan Jankowski^a*^**

^a^*Institute of Organic Chemistry, Faculty of Chemistry, Łódź University of Technology, Poland*

**^b^***Department of Neurosurgery and Peripheral Nerve Surgery, WAM University Hospital, Central Veterans Hospital of Medical University of Łódź, Poland*

*Corresponding author. Tel: +48-42-631 3222; e-mail: [stefan.jankowski@p.lodz.pl](mailto:stefan.jankowski@p.lodz.pl)

Table SM1. Concentrations of metabolites (μmol/g) in AF and NP **(control disc,** 14 years old male, L5/S1)

| Metabolite | Annulus fibrosus  m = 32,58 [mg] | Nucleus pulposus  m = 26,25 [mg] |
| --- | --- | --- |
| Isoleucine | 0.1723 | 0.1794 |
| Valine | 0.1125 | 0.1597 |
| Lactate | 1.4137 | 2.3679 |
| Alanine | 0.1440 | 0.1540 |
| Leucine | 0.1128 | 0.1138 |
| Acetate | 0.6893 | 0.6889 |
| Creatine | 4.1343 | 4.2265 |
| Glycine | 0.2436 | 0.2310 |
| Glucose | 9.5373 | 11.9247 |
| *Myo-*inositol | 1.9203 | 2.7774 |
| *Scyllo*-inositol | 2.8587 | 3.5731 |
| Taurine | 3.2649 | 3.3637 |

Table SM2. Concentrations of metabolites (μmol/g) in AF and NP **(degenerated disc,** 46 years old female, L5/S1)

| Metabolite | Annulus fibrosus  m = 29,20 [mg] | Nucleus pulposus  m = 26,20 [mg] |
| --- | --- | --- |
| Isoleucine | 2.0469 | 2.1134 |
| Valine | 1.3576 | 1.2566 |
| Lactate | 5.1712 | 2.6322 |
| Alanine | 0.1804 | 0.1912 |
| Leucine | 2.8219 | 2.7234 |
| Acetate | 1.4864 | 1.0768 |
| Creatine | 0.5332 | 0.5485 |
| Glycine | 3.3204 | 3.2270 |
| Glucose | 0.2579 | 0.2674 |
| Hydroxyproline | 4.5164 | 4.2353 |
| Chondroitin  sulfate | 4.1275 | 4.1005 |
| *Myo-*inositol | 0.1242 | 0.2604 |
| Isopropanol | 2.6188 | 1.0891 |
