# Supplementary material for: Characterization of metabolites determined by means of 1H HR MAS NMR in intervertebral disc degeneration
Source: MAGMA. 2014 Aug 10;28(2):173–83. doi: 10.1007/s10334-014-0457-0 (PMC4385564; doi:10.1007/s10334-014-0457-0)
Supplement: Supplementary file 7 — Supplementary material 7 (DOCX 15 kb) [file 10334_2014_457_MOESM7_ESM.docx]

CHARACTERIZATION OF THE METABOLITES IN INTERVERTEBRAL DISC DEGENERATION DETERMINED BY ^1^H HR MAS NMR SPECTROSCOPY

Magnetic Resonance Materials in Physics Biology and Medicine

**Barbara Pacholczyk - Sienicka^a^, Maciej Radek^b^, Andrzej Radek^b^ and Stefan Jankowski^a*^**

^a^*Institute of Organic Chemistry, Faculty of Chemistry, Łódź University of Technology, Poland*

**^b^***Department of Neurosurgery and Peripheral Nerve Surgery, WAM University Hospital, Central Veterans Hospital of Medical University of Łódź, Poland*

*Corresponding author. Tel: +48-42-631 3222; e-mail: [stefan.jankowski@p.lodz.pl](mailto:stefan.jankowski@p.lodz.pl)

Table SM3. Mean concentrations of metabolites [μmol/g] with standard deviation in intervertebral disc tissues.

| Metabolites | Control samples | Degenerated tissue  samples |
| --- | --- | --- |
| Ac | 0.2063 ± 0.0921 | 2.037 ± 1.151 |
| Acetone | 0.65 ± 0.11 | 4.59 ± 1.25 |
| Ala | 0.253 ± 0.064 | 0.23 ± 0.12 |
| Asp | 0.7041 ± 0.3905 | 1.51 ± 0.69 |
| Cit | 0.36 ± 0.14 | 0.39 ± 0.12 |
| Cr | 3.086 ± 1.239 | 0.43 ± 0.23 |
| Glc | 9.5 ± 3.2 | 1.09 ± 0.28 |
| Gly | 0.72 ± 0.62 | 3.038 ± 1.66 |
| His | 0.64 ± 0.37 | 0.92 ± 0.34 |
| Hyp | 0.339 ± 0.058 | 2.3 ± 1.5 |
| Ile | 1.0058 ± 0.6135 | 2.4 ± 1.4 |
| IPA | - | 3.6 ± 1.8 |
| Lac | 1.90 ± 0.81 | 3.2 ± 1.8 |
| Leu | 0.88 ± 0.16 | 2.5 ± 1.5 |
| Lys | 0.163 ± 0.056 | 1.88 ± 0.82 |
| MI | 4.4 ± 1.3 | 2.22 ± 1.14 |
| Formate | 0.27 ± 0.14 | 0.176 ± 0.073 |
| N-acetyl | 8.7 ± 3.7 | 2.42 ± 0.69 |
| Phe | 0.94 ± 0.47 | 1.47 ± 0.59 |
| Oxaloacetate | 0.39 ± 0.16 | 0.84 ± 0.25 |
| SI | 3.1 ± 1.9 | - |
| Suc | 0.122 ± 0.055 | 1.91 ± 0.94 |
| Tau | 6.98 ± 2.94 | - |
| Tyr | 0.47 ± 0.37 | 0.52 ± 0.21 |
| U | 0.48 ± 0.19 | 0.76 ± 0.27 |
| Val | 0.52 ± 0.45 | 1.4 ± 1.1 |
